# A novel PD-1/PD-L1 pathway molecular typing-related signature for predicting prognosis and the tumor microenvironment in breast cancer

**DOI:** 10.1007/s12672-023-00669-4

**Published:** 2023-05-08

**Authors:** Yuxin Man, Chao Dai, Qian Guo, Lingxi Jiang, Yi Shi

**Affiliations:** 1 Department of Medical Oncology, Sichuan Clinical Research Center for Cancer, Sichuan Cancer Hospital & Institute, Sichuan Cancer Center, Affiliated Cancer Hospital of University of Electronic Science and Technology of China, Chengdu, 610041 China; 2 Sichuan Provincial Key Laboratory for Human Disease Gene Study and Department of Laboratory Medicine, Sichuan Provincial People’s Hospital, University of Electronic Science and Technology of China, Chengdu, 610072 China; 3 Health Management Center, Sichuan Provincial People’s Hospital, University of Electronic Science and Technology of China, Chengdu, 610072 China; 4 Research Unit for Blindness Prevention of Chinese Academy of Medical Sciences (2019RU026), Sichuan Academy of Medical Sciences & Sichuan Provincial People’s Hospital, Chengdu, 610072 Sichuan China

**Keywords:** Breast cancer, PD-1, PD-L1, Tumor microenvironment, Signature, IFNG

## Abstract

**Background:**

Currently, the development of breast cancer immunotherapy based on the PD-1/PD-L1 pathway is relatively slow, and the specific mechanism affecting the immunotherapy efficacy in breast cancer is still unclear.

**Methods:**

Weighted correlation network analysis (WGCNA) and the negative matrix factorization (NMF) were used to distinguish subtypes related to the PD-1/PD-L1 pathway in breast cancer. Then univariate Cox, least absolute shrinkage and selection operator (LASSO), and multivariate Cox regression were used to construct the prognostic signature. A nomogram was established based on the signature. The relationship between the signature gene *IFNG* and breast cancer tumor microenvironment was analyzed.

**Results:**

Four PD-1/PD-L1 pathway-related subtypes were distinguished. A prognostic signature related to PD-1/PD-L1 pathway typing was constructed to evaluate breast cancer’s clinical characteristics and tumor microenvironment. The nomogram based on the RiskScore could be used to accurately predict breast cancer patients’ 1-year, 3-year, and 5-year survival probability. The expression of *IFNG* was positively correlated with CD8+ T cell infiltration in the breast cancer tumor microenvironment.

**Conclusion:**

A prognostic signature is constructed based on the PD-1/PD-L1 pathway typing in breast cancer, which can guide the precise treatment of breast cancer. The signature gene *IFNG* is positively related to CD8+ T cell infiltration in breast cancer.

**Supplementary Information:**

The online version contains supplementary material available at 10.1007/s12672-023-00669-4.

## Introduction

As one of the most common cancers worldwide, breast cancer is the leading cause of tumor-related death in women [1]. Currently, treatment strategies for breast cancer are selected based on histopathology and specific genetic characteristics, mainly including surgery, hormone replacement, chemoradiotherapy, and targeted therapy [2, 3]. However, the current effect of conventional treatment is not ideal for patients with advanced breast cancer, especially those with triple-negative breast cancer [4].

As critical immune checkpoints, PD-1/PD-L1 can regulate the function of T cells and play an essential role in maintaining immune homeostasis under physiological conditions [5, 6]. In the complex tumor microenvironment, the PD-1/PD-L1 pathway controls immune tolerance in the tumor microenvironment, which is the main mechanism of immune escape of tumor cells [7]. In recent years, immune checkpoint inhibitors targeting the PD-1/PD-L1 pathway have shown great success and promoted the development of immunotherapy in various tumors, bringing hope to breast cancer.

Compared with lung cancer, malignant melanoma, breast cancer has a low gene mutation rate and poor immunogenicity and was previously considered a cold immune tumor [8, 9]. As the clinical practice of PD-1/PD-L1 inhibitors in breast cancer is in full swing and the understanding is deepening, more and more clinical trials such as KEYNOTE-119 [10] and NEWBEAT [11] have confirmed the close connection between breast cancer and immunotherapy. However, breast cancer, a complex genetic disease, can be further subdivided into luminal A, luminal B, HER2-positive, and basal-like. It has the characteristics of strong heterogeneity and significant individual differences. Prognosis among different subtypes and tumor immune-related indicators such as tumor-infiltrating lymphocytes, PD-L1 expression, and tumor mutation burden (TMB) are pretty different [12]. Currently, PD-1/PD-L1 inhibitors have limited efficacy in treating hormone receptor-positive metastatic breast cancer. Even in triple-negative breast cancer with the most popular immunotherapy research, the objective response rate of single-agent PD-1/PD-L1 inhibitor therapy is difficult to more than 30% [9]. There are many unknowns about the PD-1/PD-L1 pathway in breast cancer, and the overall response rate of PD-1/PD-L1 inhibitors in patients is not ideal, which limits its clinical application. It is urgent to clarify further the relationship between the PD-1/PD-L1 pathway and clinical characteristics of breast cancer patients to carry out risk stratification and guide precise clinical treatment.

**Fig. 1:**
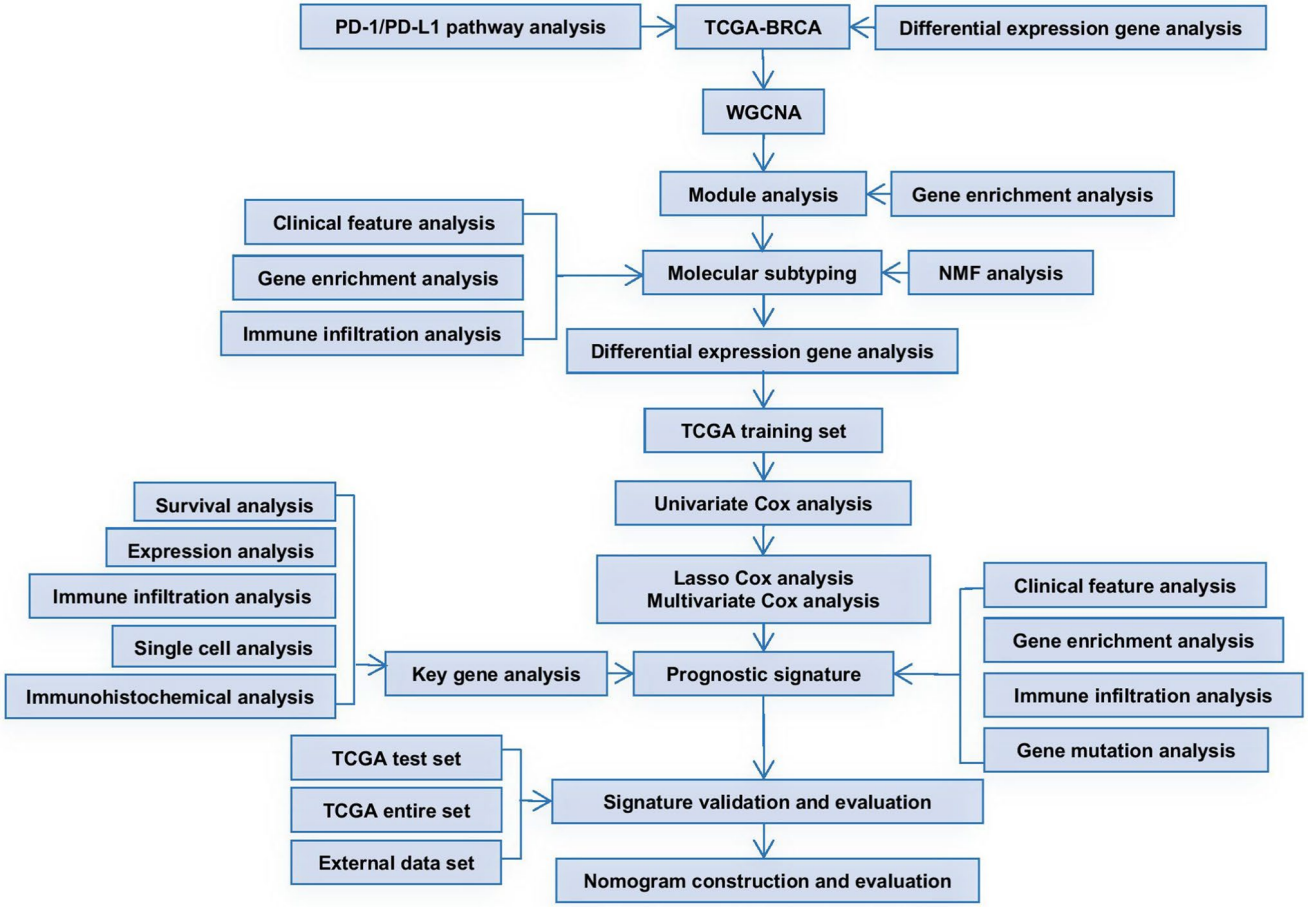
The study flowchart. Firstly, WGCNA was performed on breast cancer samples in the TCGA database, and a total of 25 key genes of the PD-1/PD-L1 pathway in breast cancer were identified. TCGA breast cancer samples were classified accordingly. Next, univariate Cox analysis, LASSO, and multivariate Cox analysis were used to screen prognostic genes. A seven-gene signature was constructed, and the nomogram was further built. Subsequently, immune correlation analysis, somatic mutation, drug sensitivity analysis, GO, KEGG, and GSEA were applied to determine the clinical applicability of this signature. Finally, immunohistochemistry was used to verify the expression and function of the key prognostic gene

In recent years, the era of big medical data has made it possible for us to conduct multi-omics and multi-angle analyses of tumors to find biomarkers [13–15]. We systematically evaluated the characteristics of the PD-1/PD-L1 pathway in breast cancer, based on which we performed molecular classification of breast cancer and analyzed the clinical prognostic characteristics of different subtypes. The prognostic signature related to PD-1/PD-L1 pathway molecular typing was further constructed, and a nomogram was drawn to predict the survival of breast cancer patients. Immunohistochemistry was used to verify the expression of the signature gene *IFNG* in breast cancer samples and we further explored the relationship between *IFNG* and immune infiltrating cells in the breast cancer tumor microenvironment. The workflow of this study is shown in Fig. 1.

## Materials and methods

### Data acquisition and differentially expressed genes (DEGs) analysis

TCGA-BRCA (breast cancer) and breast tissue protein coding gene data were obtained from the TCGA database, and DESeq2 [16] was used for data processing. GSE20711 [17], GSE103091 [18], GSE42568 [19], and GSE76250 [20] were obtained from the GEO database, and limma [21] was used for data processing. DEGs were defined as the adjusted *P* value < 0.05, |log_2_foldchange| > 1. The public immunohistochemical staining information was obtained from the HPA database [22]. Breast cancer single-cell sequencing data were obtained from the single-cell sequencing dataset in Single Cell Portal (https://singlecell.broadinstitute.org/single_cell) [23]. The “PD-L1 expression and PD-1 checkpoint pathway in cancer” gene set (hsa05235) was downloaded from the Kyoto protocol encyclopedia of genes and genomes (KEGG) database, and the gene set was presented in the supplementary materials (Supplementary Table S1).

### PD-1/PD-L1 pathway scoring and function enrichment analysis

The PD-1/PD-L1 pathway in breast cancer was analyzed by gene set variation analysis (GSVA) [24]. Gene ontology (GO) or KEGG analysis was performed using “ClusterProfiler” [25]. “h.all.v7.5.1 symbols.gmt” was obtained from MSigDB database for gene set enrichment analysis (GSEA) [26].

### Establishment of WGCNA and identification of the key module related to the PD-1/PD-L1 pathway in breast cancer

DEGs were included in WGCNA [27] to identify the module most related to the PD-1/PD-L1 pathway in breast cancer. The soft threshold β was selected when close to 0.9 (1 to 20). The hierarchical clustering method was used to identify modules (the minimum number of genes in the module was 30), and similar modules were merged (abline = 0.25). Pearson correlation analysis was used to evaluate the correlation between module eigengene (ME, the first principal component of a given module, representing the gene expression of the whole module) and PD-1/PD-L1 pathway scores. Gene significance (GS) represented the Pearson correlation coefficient between gene expression and the PD-1/PD-L1 pathway. Modular membership (MM) could be obtained by correlation analysis between the expression level of this gene and the module eigengene. MM was used to measure the importance of genes in the module. GS greater than 0.5 and MM greater than 0.8 were selected to screen genes in the module.

### Molecular typing based on the PD-1/PD-L1 pathway in breast cancer

TCGA breast cancer samples were classified by non-negative matrix factorization (NMF) (method = “brunet”) [28] according to breast cancer PD-1/PD-L1 pathway key genes obtained in the above steps. The appropriate subtype fraction number k was determined by cophenetic correlation, dispersion, and silhouette.

### Assessment of tumor microenvironment in breast cancer samples

ESTIMATE [29], ImmuneCellAI (http://bioinfo.life.hust.edu.cn/ImmuCellAI#!/) [30], CIBERSORTx (https://cibersortx.stanford.edu/) [31], EaSIeR [32], and TIMER2.0 (http://timer.cistrome.org/) [33] were applied to analyze the tumor microenvironment in breast cancer.

### Development of the PD-1/PD-L1 pathway molecular typing-related prognostic signature

Univariate Cox analysis was performed in the training set, and results with *P* < 0.01 were included in least absolute contraction and selection operator (LASSO) regression to identify PD-1/PD-L1 pathway-related prognostic genes. The prognostic signature was further constructed by the multivariate Cox regression survival analysis step method. *Coefj* represented the coefficient, and *Xj* represented the normalized gene expression. *h*_*0*_* (t)* was the baseline hazard function. The signature’s predictive ability was quantified by the area under the curve (AUC) and C-index. The RiskScore was calculated using the “predict” function (type = “risk”) in the R software by the following formula:

$$RiskScore:h_{0} \left( t \right)\exp \left( {\sum_{j = 1}^{n} Coefj*Xj} \right).$$

### Clinical features analysis

“maftools” [34] was used to analyze TCGA-BRCA gene mutation data. “oncoPredict” [35, 36] was used to predict IC_50_ of common antitumor drugs with different RiskScores and Spearman correlation was used to analyze the relationship between the RiskScore and the drug IC_50_.

### Construction of the nomogram

The nomogram was constructed by “regplot” in combination with important clinical parameters of breast cancer. The clinical predictive ability of this nomogram was evaluated using C-index, the calibration curve, and decision curve analysis (DCA).

### Immunohistochemical analysis

Tissue microarray (TMA) (HBreD050Bc01) was obtained from Shanghai Outdo Biotech Company (Shanghai, China). Anti‐rabbit IFNG antibody (15365-1-AP) was obtained from Proteintech, and the working concentration was 1:8000. The immunohistochemical staining process of TMA was shown before [37, 38]. The staining intensity (0/1+/2+/3+) and positive rate (staining positive rate score: 0–100% corresponding to 0–100 points) of IFNG in cytoplasm and nucleus were interpreted. Cancer tissues and adjacent tissues were analyzed respectively. “Staining intensity” and “staining positive rate” were multiplied to give the final sample score.

### Statistical analysis

Statistical analysis and drawing were performed with R v.4.2.2, SPSS 26, and GraphPad Prism 9. Student’s *t-test* or Wilcoxon test was used to compare the two groups. One-way ANOVA or Kruskal–Wallis test was used to compare multiple groups. Survival analysis was performed using the log-rank test, and *P* < 0.05 was considered statistical significance.

## Results

### Analysis of the PD-1/PD-L1 pathway-related DEGs in breast cancer

**Fig. 2:**
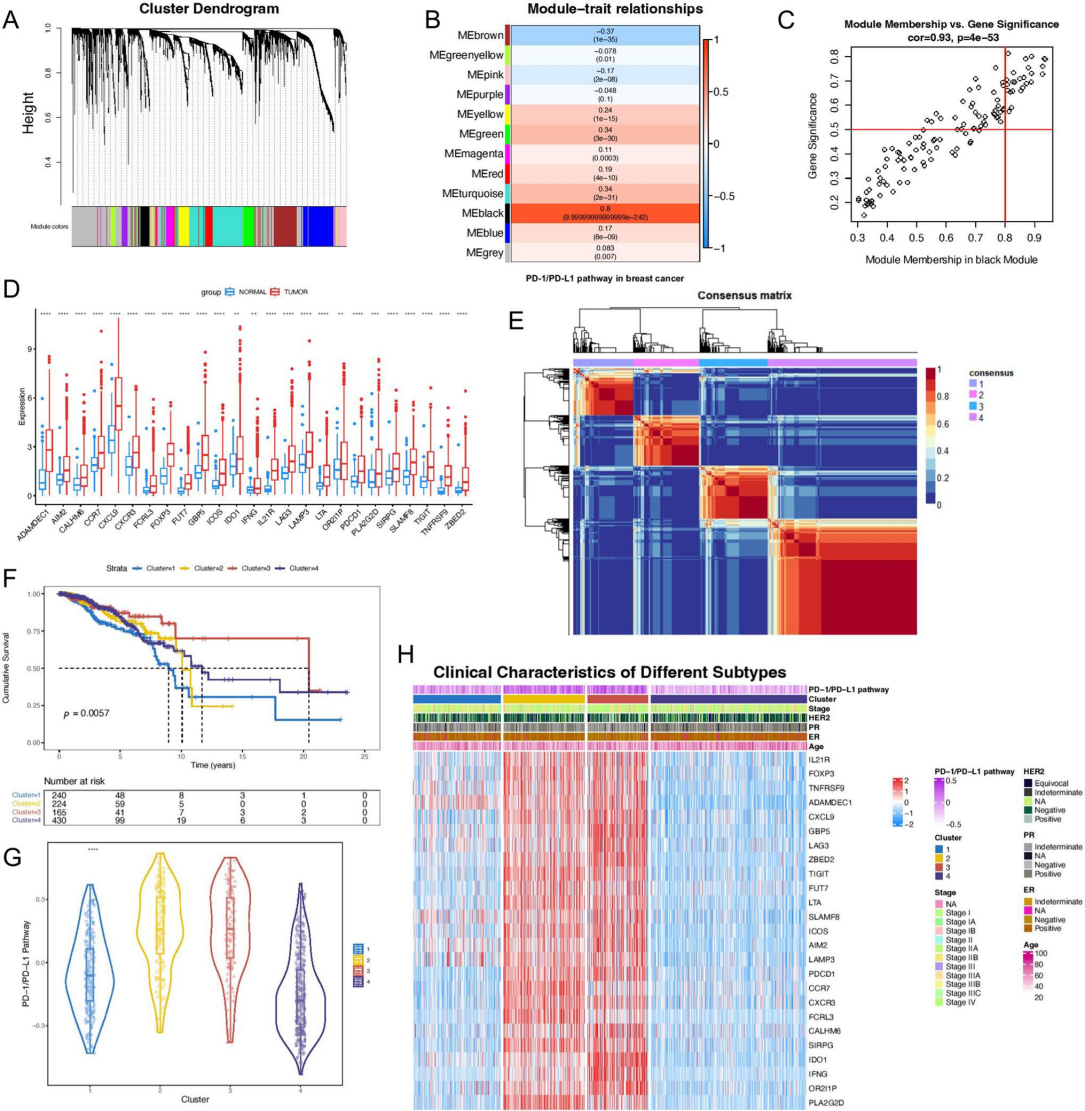
The PD-1/PD-L1 pathway molecular subtype analysis. **A** Hierarchical clustering dendrogram of genes; **B** Module-phenotypic correlation analysis. Each cell contained the corresponding correlation and *P* value; **C** GS and MM correlation scatter diagram in the black module. GS was highly significantly correlated with MM. Results suggested that genes highly associated with the PD-1/PD-L1 pathway were also important in the black module; **D** The box diagram of expression of 25 key genes in TCGA breast cancer and normal tissues; **E** The consensus heatmap of NMF; **F** Survival analysis of different breast cancer subtypes in TCGA; **G** The violin plot of four subtypes of PD-1/PD-L1 pathway scores; **H** The heatmap of the relationship between characteristic gene expression and clinical parameters of different molecular subtypes of breast cancer in the TCGA database. ****P < 0.0001; **P < 0.01

As a widely used database, the TCGA database contains a large sample size of breast cancer samples. This study was based on the TCGA database. Firstly, differential expression of coding genes was analyzed between 1076 breast cancer samples and 99 breast samples in the TCGA database. The results indicated that a total of 5090 genes were differentially expressed. Due to the excessive number of DEGs, the |log_2_foldchange| was further increased to 1.5, resulting in a total of 2973 genes. The expression data of these 2973 genes were used to construct the breast cancer-related weighted co-expression network. When the soft threshold β was 4 (Fig. S1A), the constructed weighted co-expression network was closest to the scale-free network. By GSVA, the PD-1/PD-L1 pathway score of each breast cancer sample was obtained and included in WGCNA for correlation analysis. Twelve modules were obtained by clustering, represented by different colors (Fig. 2A).

According to the correlation heatmap (Fig. 2B), the black module had the most significant correlation with the PD-1/PD-L1 pathway in breast cancer samples, with a correlation coefficient of 0.8 (*P* < 0.01). KEGG (Fig. S1B) analysis of modules suggested that they were involved in many critical biological functions of immunology, such as cytokine and cytokine receptor interaction, Toll-like receptor signaling pathway, and chemical factor signaling pathway. Therefore, the study focused on the black module (120 DEGs). In the black module, GS was positively correlated with MM (correlation coefficient was 0.93) (Fig. 2C), indicating that this module was closely related to the PD-1/PD-L1 pathway. According to the preset thresholds of GS and MM, 25 DEGs in the black module had the strongest correlation with the PD-1/PD-L1 pathway in breast cancer, and it suggested that these 25 genes were closely related to cytokines and T cell function (KEGG analysis) in the PD-1/PD-L1 pathway and might be the essential genes related to the PD-1/PD-L1 pathway in breast cancer.

### Identification of the PD-1/PD-L1 pathway-related molecular subtypes in breast cancer

After the above steps, 25 key genes of the PD-1/PD-L1 pathway in breast cancer were found. The expression of these 25 genes was analyzed in the TCGA database (Fig. 2D). It was known that they were up-regulated in breast cancer samples compared to breast tissues. These 25 genes might play an essential role in the occurrence and development of breast cancer. We used the NMF method to type TCGA breast cancer samples (n = 1059) based on these 25 key genes. By analyzing cophenetic correlation, dispersion, and silhouette, line plots showed a significant degree of inflection point at k = 4, so the optimal cluster number in this study was 4 (Fig. S1C). Four molecular subtypes [Cluster 1 (n = 240), Cluster 2 (n = 224), Cluster 3 (n = 165) and Cluster 4 (n = 430)] differentiated by the PD-1/PD-L1 pathway were obtained. The clinical data of TCGA breast cancer samples were shown in Table 1. The consistency clustering was shown in Fig. 2E. Principal component analysis (PCA) of molecular subtypes (Fig. S1D) showed that key genes of the PD-1/PD-L1 pathway could effectively classify TCGA breast cancer samples. Survival analysis among different molecular subtypes indicated that survival with different molecular subtypes was significantly different (*P* < 0.01) (Fig. 2F). We also found significant differences in PD-1/PD-L1 pathway scores among the four subtypes (Fig. 2G). These results suggested that our molecular typing of TCGA breast cancer samples based on these 25 genes had crucial clinical significance.

**Table 1:** Clinical parameters of patients in the TCGA-BRCA cohort

Clinical parameters	TCGA-BRCA
No. of patients	1059
Age [mean (SD)]	58.35 (13.18)
ER (%)	
Negative	232 (21.9)
Positive	780 (73.7)
Indeterminate	2 (0.2)
NA	45 (4.2)
PR (%)	
Negative	330 (31.2)
Positive	679 (64.1)
Indeterminate	4 (0.4)
NA	46 (4.3)
HER2 (%)	
Negative	542 (51.2)
Positive	157 (14.8)
Equivocal	176 (16.6)
Indeterminate	11 (1.0)
NA	173 (16.3)
Stage (%)	
Stage I	87 (8.2)
Stage IA	84 (7.9)
Stage IB	6 (0.6)
Stage II	6 (0.6)
Stage IIA	347 (32.8)
Stage IIB	243 (22.9)
Stage III	2 (0.2)
Stage IIIA	153 (14.4)
Stage IIIB	25 (2.4)
Stage IIIC	64 (6.0)
Stage IV	20 (1.9)
NA	22 (2.1)
Cluster (%)	
1	240 (22.7)
2	224 (21.2)
3	165 (15.6)
4	430 (40.6)

In order to show the characteristics of each cluster. The clinical parameters (PD-1/PD-L1 pathway, Subtype, Stage, Age, ER/PR/HER2 status) and the heatmap of 25 key genes of different subtypes were further drawn (Fig. 2H) (Supplementary Table S2). Through the heatmap, we could observe that these 25 genes had significant differences in expression among the four subtypes, suggesting that there might be different biological characteristics. At the same time, gene mutation waterfall maps among different molecular subtypes of breast cancer were drawn (Fig. S2). Cluster 1 and Cluster 3 were dominated by *TP53* mutation, while Cluster 2 and Cluster 4 were dominated by *PIK3CA* mutation.

### Analysis of molecular mechanism and immunological characteristics among subtypes

According to “h.all.v7.5.1 symbols.gmt”, hallmark pathways of different breast cancer molecular subtypes were scored, and the heatmap was drawn (Fig. S1E). It could be observed that the scores of MTORC1 SIGNALING and UNFOLDED PROTEIN RESPONSE in Cluster 1 were high. In Cluster 2, pathways such as INFLAMMATORY RESPONSE had higher scores. Pathways such as REACTIVE OXYGEN SPECIES PATHWAY had higher scores in Cluster 3. Pathways such as ESTROGEN RESPONSE were scored higher in Cluster 4.

**Fig. 3:**
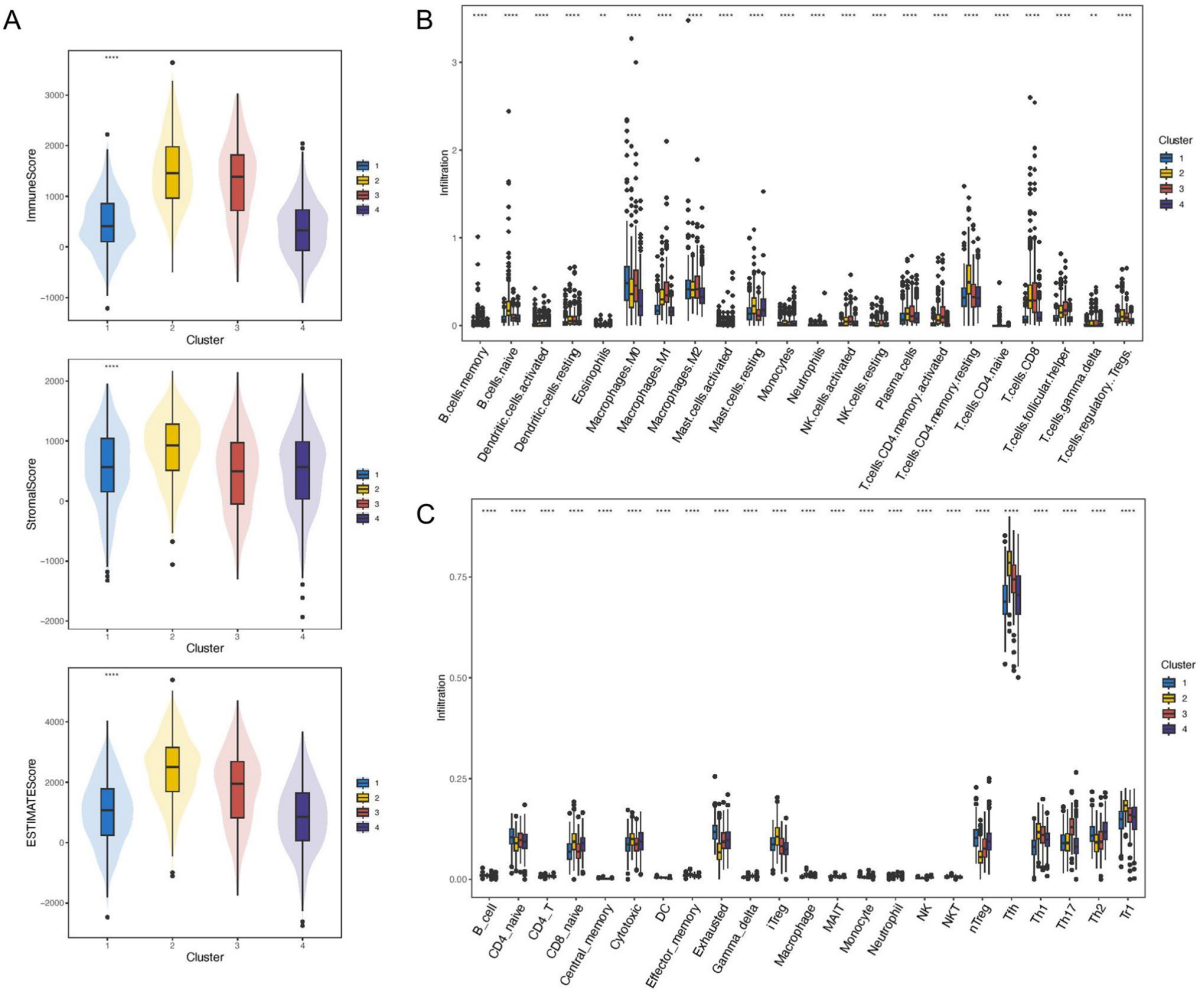
Tumor microenvironment analysis of different molecular subtypes. **A** ESTIMATE analysis of different molecular subtypes of breast cancer in TCGA database; **B** CIBERSORTx analysis of different molecular subtypes of breast cancer in TCGA database; **C** ImmuCellAI analysis of different molecular subtypes of breast cancer in TCGA database. ****P < 0.0001; ***P < 0.001; **P < 0.01; *P < 0.05

Differences in the tumor microenvironment of each subtype were further analyzed, and the tumor microenvironment was evaluated by ImmuneScore, StromalScore, and ESTIMATEScore (Fig. 3A). ImmuneScore in Cluster 2 and Cluster 3 was relatively high, indicating more immune-related components in the tumor microenvironment. In CIBERSORTx (Fig. 3B) and ImmuCellAI (Fig. 3C) results, tumor microenvironment components differed significantly among subtypes. M0 macrophages were significantly infiltrated in Cluster 1. Tfh cells were significantly infiltrated in Cluster 2, and M1 macrophages were significantly infiltrated in Cluster 3. These results suggested that molecular subtypes of breast cancer differentiated by the PD-1/PD-L1 pathway significantly differed in pathogenesis and the tumor microenvironment.

### Identification of key genes related to molecular typing of the PD-1/PD-L1 pathway in breast cancer

The above steps analyzed clinical survival and tumor microenvironment characteristics of different subtypes distinguished by the PD-1/PD-L1 pathway in breast cancer. Cluster 1 and Cluster 3 showed the most considerable difference in prognosis, and there was a significant difference in ImmuneScore between Cluster 1 and Cluster 3, suggesting that genes that influenced the tumor microenvironment and prognosis were most differentially expressed between the two subtypes. Therefore, we further investigated changes in 2973 DEGs between Cluster 1 and Cluster 3 (Supplementary Table S3). Between Cluster 1 and Cluster 3, 246 genes were differentially up-regulated, and 257 were differentially down-regulated. Similarly, 219 DEGs with the absolute value of log_2_foldchange greater than 1.5 were included in the subsequent analysis.

### Construction and validation of the PD-1/PD-L1 pathway molecular typing-related prognostic signature

**Fig. 4:**
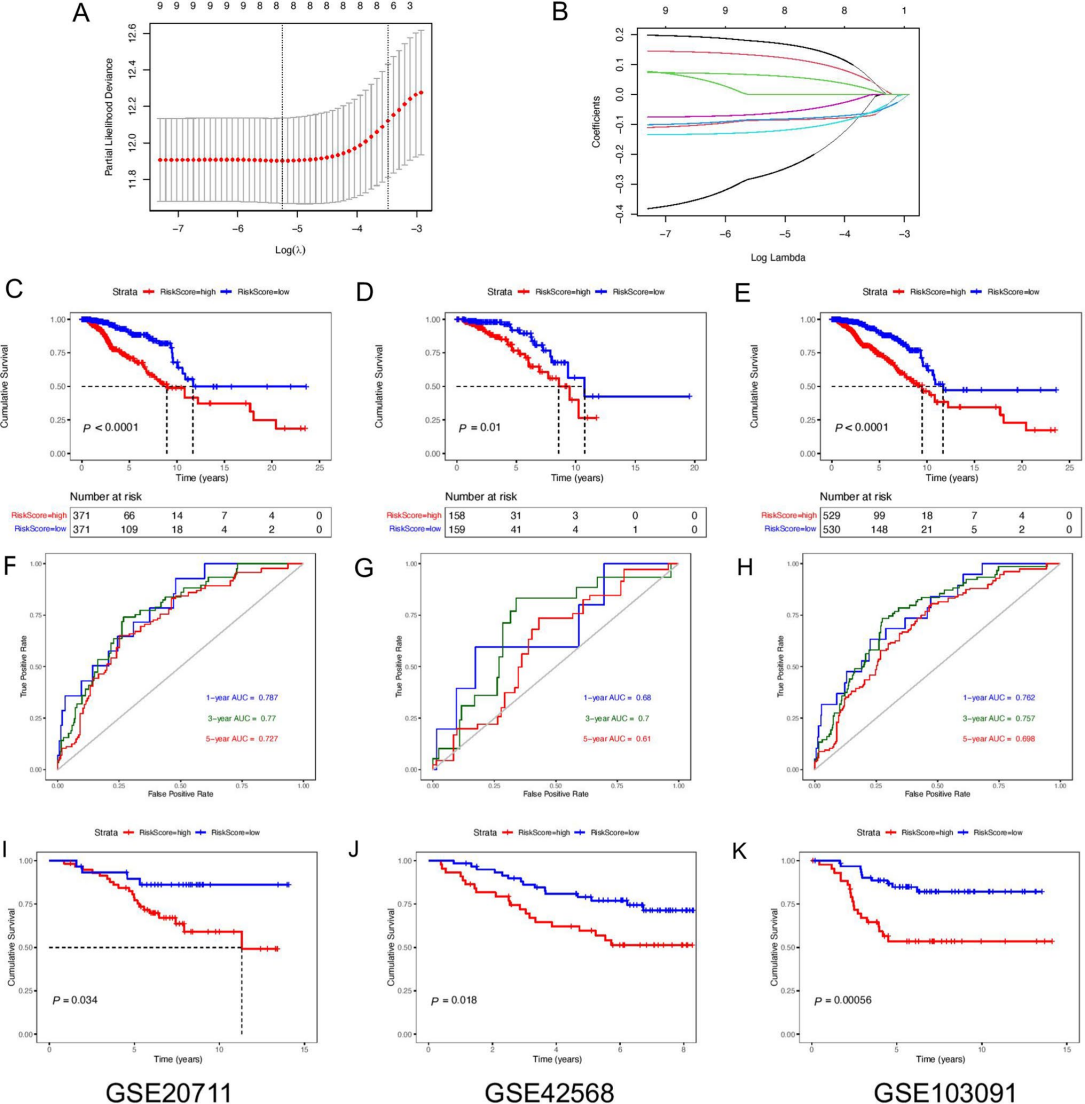
Construction of the prognostic signature. **A** Ten-fold cross-validation of λ selection in LASSO analysis; **B** LASSO coefficient spectrum; **C**–**E** TCGA breast cancer samples were randomly divided into the training set and the internal test set at a 7:3 ratio. Survival analysis showing the difference in the prognosis of the training set, the internal test set, and the entire set in high- and low-RiskScore group, respectively; **F**–**H** 1-, 3- and 5-year ROC curves of the training set, the internal test set, and the entire set, respectively; **I** Survival analysis in the prognosis of GSE20711 in high- and low-RiskScore group; **J** Survival analysis in the prognosis of GSE42568 in high- and low-RiskScore group; **K** Survival analysis in the prognosis of GSE103091 in high- and low-RiskScore group

In order to better construct the prognostic signature related to the molecular typing of the PD-1/PD-L1 pathway, the breast cancer samples with prognostic follow-up time greater than 0 in the TCGA database were randomly divided into the training set (Supplementary Table S4) and the internal test set (Supplementary Table S5) according to the ratio of 7:3. Univariate Cox analysis was performed on the 219 DEGs obtained from the molecular classification in the above steps (Supplementary Table S6). According to the cut-off value of *P* < 0.01, nine genes were associated with breast cancer prognosis in the training set and were used as candidates to establish the LASSO model. Subsequently, to obtain the critical prognostic genes, LASSO analysis was performed on these nine genes, in which lambda = 0.0063 (Fig. 4A, B), and eight genes were included in multivariate Cox regression analysis. Finally, seven non-collinear genes related to the PD-1/PD-L1 pathway molecular typing were selected to construct the prognosis signature: *IFNG*, *JCHAIN*, *ELOVL2*, *PIGR*, *PAGE5*, *ACTL8*, and *CLEC3A*. The PD-1/PD-L1 pathway molecular typing-related prognostic signature was calculated using the “predict” function (type = “risk”) in the R software:

$$\begin{aligned} \sum_{j = 1}^{n} Coefj*Xj: & - 0.4069 \times IFNG - 0.0894 \times JCHAIN - 0.1312 \times ELOVL2 - 0.0926 \times PIGR \\ & \quad + 0.2100 \times PAGE5 + 0.1393 \times ACTL8 + 0.0797 \times CLEC3A. \\ \end{aligned}$$

In the TCGA dataset, univariate and multivariate Cox regression analyses were performed on relevant clinical variables, and both showed that RiskScore was significantly correlated with survival (*P* < 0.01). Results were shown in Table 2, indicating the independence of this signature in clinical application.

**Table 2:** Univariate and multivariate Cox analyses

Clinical parameters	Univariable Cox				Multivariable Cox			
	HR	95% CI of HR		*P*	HR	95% CI of HR		*P*
		Lower	Upper			Lower	Upper	
Age	1.04	1.03	1.06	< 0.01	1.04	1.02	1.06	< 0.01
ER	0.64	0.39	1.06	0.083	–	–	–	–
PR	0.73	0.46	1.17	0.191	–	–	–	–
HER2	1.72	1.04	2.84	0.035	1.34	0.79	2.27	0.29
Stage	2.85	1.79	4.52	< 0.01	3.33	2.06	5.37	< 0.01
RiskScore	1.23	1.16	1.31	< 0.01	1.21	1.13	1.30	< 0.01

Subsequently, in the TCGA cohort, survival was assessed using the Kaplan–Meier method. Datasets were grouped according to the median RiskScore. The prognostic signature was validated in the training set, the internal test set, and the entire set. In the training set, survival analysis suggested that patients with low-RiskScore had significantly better survival outcomes (Fig. 4C–E). The C-index was 0.747. AUC values indicated that the signature had the predictive ability (1-year AUC = 0.787; 3-year AUC = 0.770; 5-year AUC = 0.727) (Fig. 4F). This signature also showed good prediction ability in the internal test set and the entire set (Fig. 4G, H).

In the TCGA breast cancer samples, we further analyzed the impact of the RiskScore on patient survival in different age, ER/PR/HER2 status, and tumor stage. The results showed that the low-RiskScore patients had the better prognosis according to “surv_cutpoint” function (Fig. S3). GEO datasets from different sources (GSE20711, GSE42568, and GSE103091) were used as the external validation sets. A survival advantage was also observed for patients with low-RiskScore according to “surv_cutpoint” function (Fig. 4I–K). Also, we analyzed whether there were differences in the RiskScore among different subtypes of breast cancer (Fig. S4), and the results indicated that there were significant differences in the RiskScore among different subtypes. The RiskScore of Cluster 1 was relatively high, which was consistent with the poor prognosis.

### Clinical features with different RiskScores

**Fig. 5:**
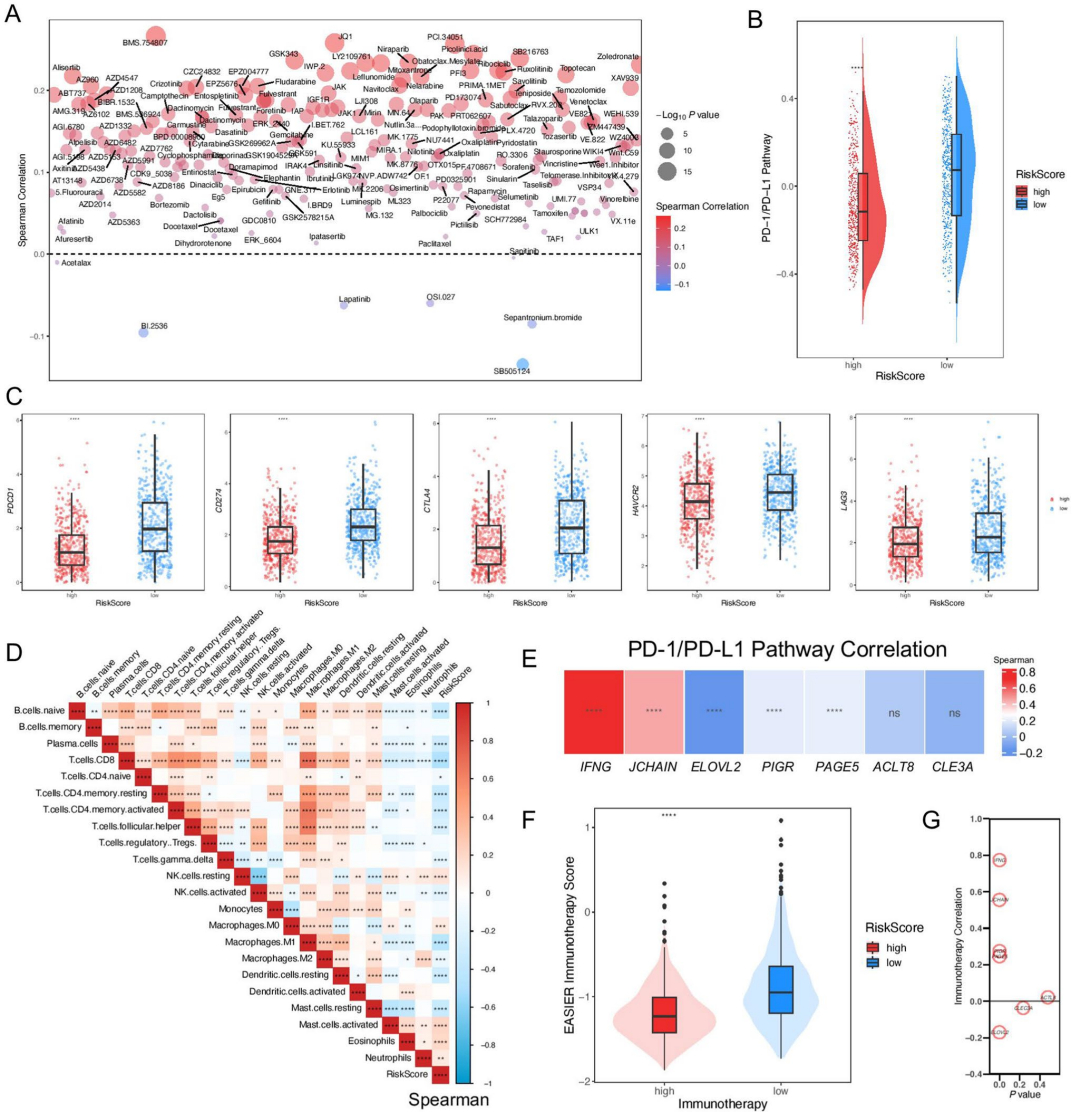
Analysis of the clinical application of the prognostic signature. **A** The bubble plot of correlation between RiskScores and IC_50_ values of antineoplastic agents; **B** Differences in PD-1/PD-L1 pathway scores of patients with different RiskScores; **C** Expression of *PDCD1*, *CD274*, *CTLA-4*, *HAVCR2*, and *LAG3* in different RiskScore groups; **D** CIBERSORTx predicted tumor microenvironment in different RiskScore patients; **E** The heatmap of correlation between signature genes and the PD-1/PD-L1 pathway; **F** Differences in RiskScores for response to the immunotherapy; **G** The bubble map of correlation between signature genes and immunotherapy. ****P < 0.0001; ***P < 0.001; **P < 0.01; *P < 0.05; *ns* no significance

The clinical characteristics of breast cancer patients in different groups in the TCGA dataset were further analyzed. Firstly, IC_50_ values of antitumor drugs were predicted (Fig. 5A), and the mechanism of each antitumor drug was described in Supplementary Table S7. We further analyzed the relationship between the RiskScore and IC_50_ of antineoplastic drugs. The results showed that the IC_50_ of most drugs increased with the increase of the RiskScore, suggesting that the RiskScore could also guide the precise treatment of patients in clinical practice. Since the prognostic signature was constructed by the PD-1/PD-L1 pathway typing, we first evaluated the differences of the PD-1/PD-L1 pathway in patients with different RiskScores. The result showed that PD-1/PD-L1 pathway scores were significantly increased in patients with low RiskScores (Fig. 5B). we further analyzed the immune-related characteristics of patients with different RiskScores. The expression levels of *PDCD1*, *CD274*, *CTLA-4, HAVCR2,* and *LAG3* were relatively higher in the low-RiskScore group (Fig. 5C). CIBERSORTx was used to analyze the tumor microenvironment of patients with different RiskScores (Fig. 5D). According to the results, CD8-T cells, activated NK cells, and M1 macrophages were clearly negatively correlated with the RiskScore, and the results suggested significant differences in the infiltration of different cells in the tumor microenvironment between the two groups. We also analyzed the correlation between each indicator gene and the PD-1/PD-L1 pathway, and found that *IFNG* was significantly positively correlated with the PD-1/PD-L1 pathway, with a correlation coefficient greater than 0.7 (*P* < 0.05) (Fig. 5E). We also predicted the response to immunotherapy in breast cancer samples with different RiskScores, and the low-RiskScore group had a higher immunotherapy score (Fig. 5F). Interestingly, *IFNG* also showed a significantly positive correlation with immunotherapy compared with other signature genes (Fig. 5G). The above results suggested that subsequent studies might need to focus on the relationship between *IFNG* and breast cancer microenvironment.

The gene mutation also completely differed between groups (Fig. S5A). In the high-RiskScore group, mutation frequencies of *TP53*, *PIK3CA*, *TTN*, *GATA3*, and *MUC16* were 41%, 34%, 19%, 11%, and 11%, respectively. In the low-RiskScore group, mutation frequencies of *PIK3CA*, *TP53*, *TTN*, *CDH1*, and *GATA3* were 35%, 27%, 19%, 18%, and 13%, respectively. Immunohistochemical staining in cancer tissues was obtained from the HPA database, and the staining distribution of IFNG, JCHAIN, PIGR, PAGE5, and CLEC3A in cancer tissues could be preclinically observed in Fig. S5B.

### Construction of the nomogram

**Fig. 6:**
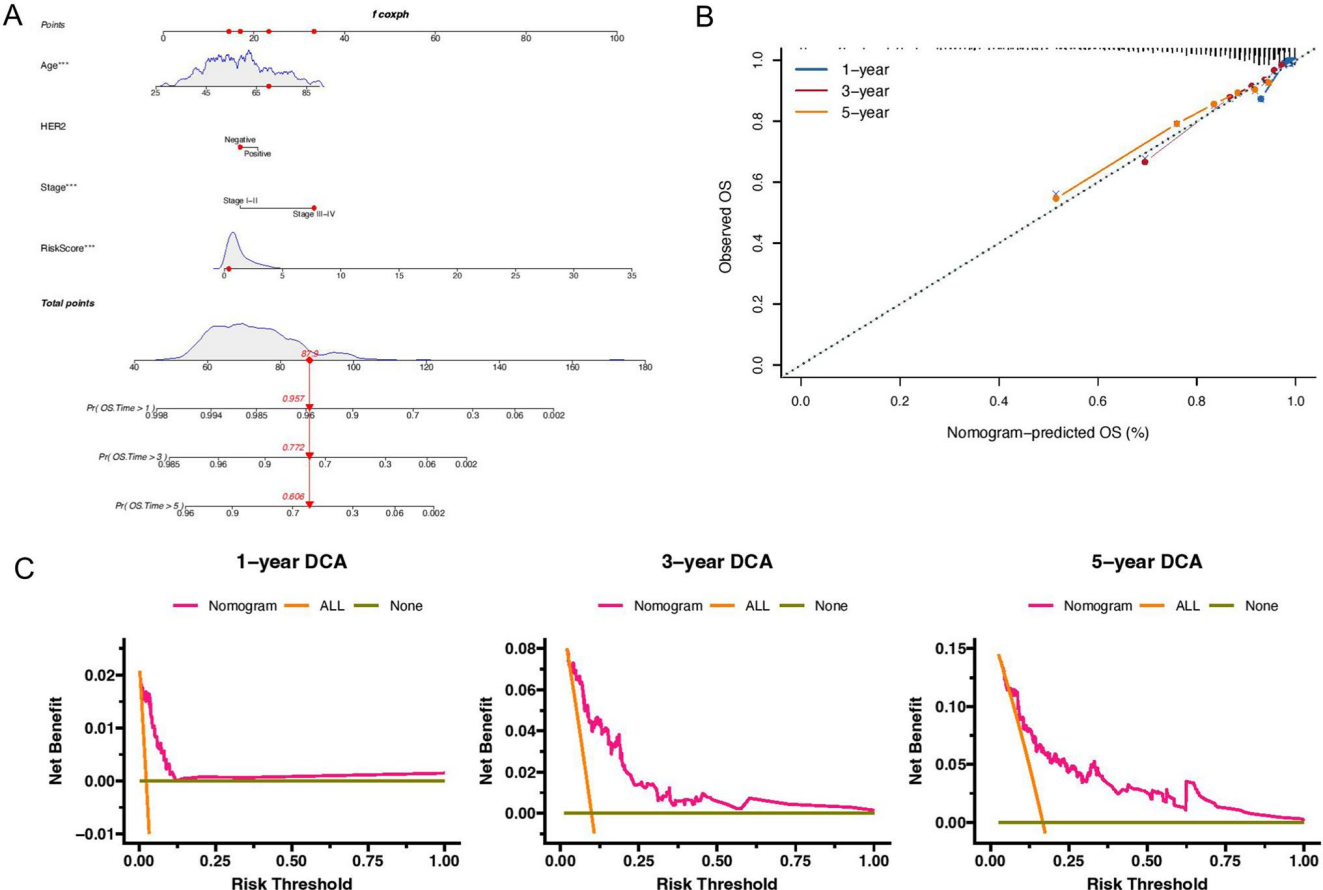
Construction of the nomogram. **A** The nomogram was constructed by combining different clinical parameters. **B** Calibration curves, which could be used to show the actual survival probability and the predicted probability; **C** DCA, which could be used to evaluate the clinical utility of the nomogram. ***P < 0.001

Based on the PD-1/PD-L1 pathway typing-related prognostic signature, the nomogram was constructed by multivariate Cox regression analysis of Age, HER2, Stage, and RiskScore (Fig. 6A). For example, a 70-year-old female patient with ER/PR/HER2 negative, stage III-IV, and RiskScore of 0.392 could be calculated by the nomogram, and the final total score was 87.9. The probability of survival over 1 year, 3 years and 5 years was 0.957, 0.772, and 0.606, respectively. The C-index of this nomogram was 0.812 (se = 0.027), and the calibration curve (Fig. 6B) and DCA (Fig. 6C) proved that the nomogram had reasonable clinical practicability for prognostic prediction.

### Mechanism analysis of the RiskScore

**Fig. 7:**
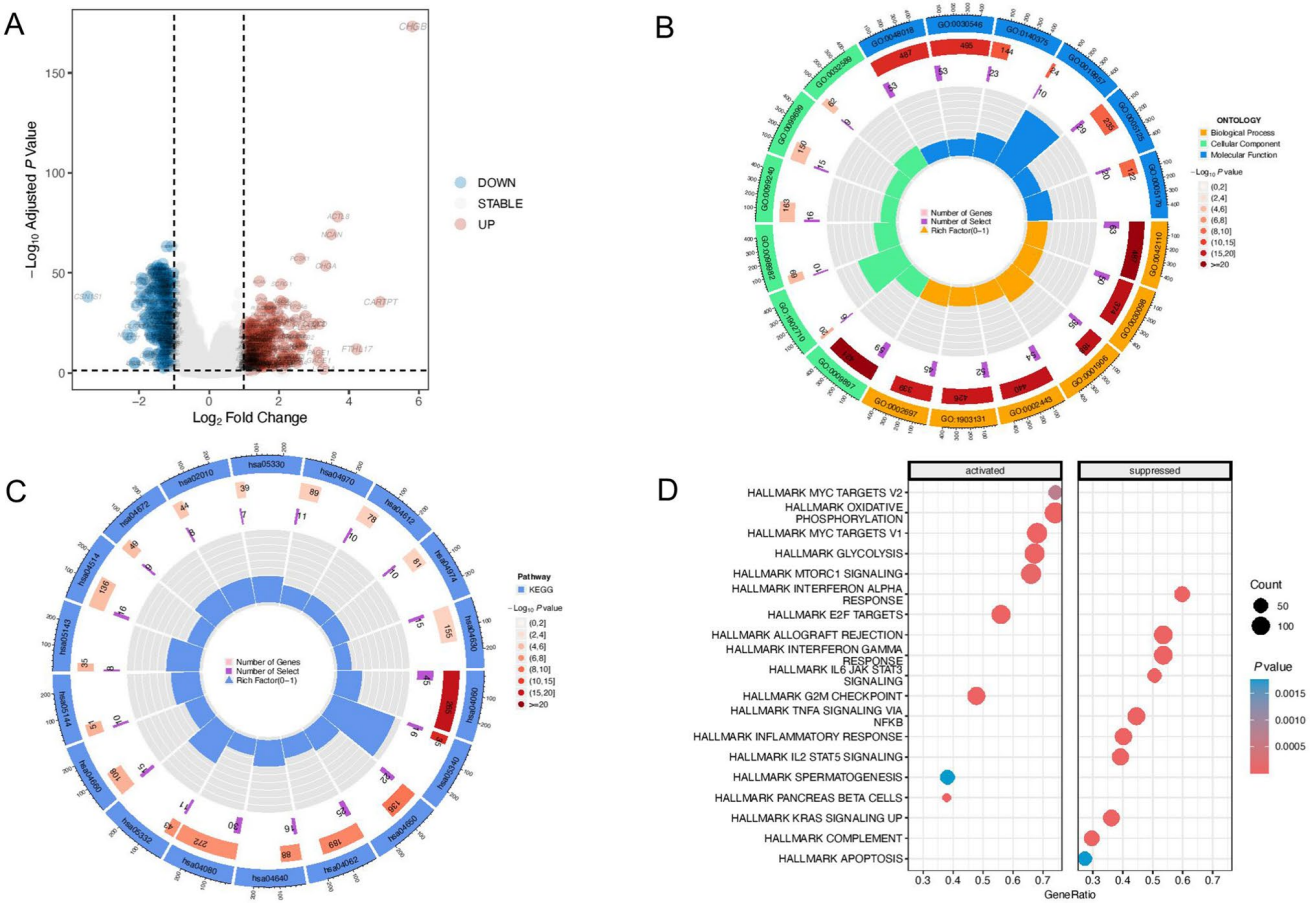
Exploration of molecular mechanisms between different RiskScore groups in the TCGA database: **A** The volcano plot: RiskScore-related DEGs in TCGA-BRCA samples; **B** GO analysis of RiskScore-related DEGs; **C** KEGG analysis of RiskScore-related DEGs; **D** GSEA of RiskScore-related DEGs

The above research showed significant differences in clinical prognosis, gene mutation spectrum, and tumor microenvironment immune infiltration between the two groups. Therefore, we further explored the molecular mechanism between the two groups. DEGs of the high- and low-RiskScore groups in the TCGA dataset were explored in depth (Fig. 7A) (Supplementary Table S8). It could be seen that 348 genes were up-regulated and 351 genes were down-regulated due to the difference in RiskScores. The possible primary mechanism of the RiskScore affecting the clinical immunophenotype of breast cancer was explored. We performed GO (Fig. 7B) (Supplementary Table S9), KEGG (Fig. 7C) (Supplementary Table S10), and GSEA (Fig. 7D) analyses. According to KEGG, the RiskScore mainly affected cytokine-cytokine receptor interaction, primary immunodeficiency, and natural killer cell-mediated cytotoxicity. According to GSEA, high-RiskScore activated OXIDATIVE PHOSPHORYLATION, E2F TARGETS, G2M CHECKPOINT, and GLYCOLYSIS, and it suggested that APOPTOSIS and INFLAMMATORY RESPONSE were suppressed.

### Relationship between *IFNG* and CD8+ T cell infiltration in breast cancer tumor microenvironment

**Fig. 8:**
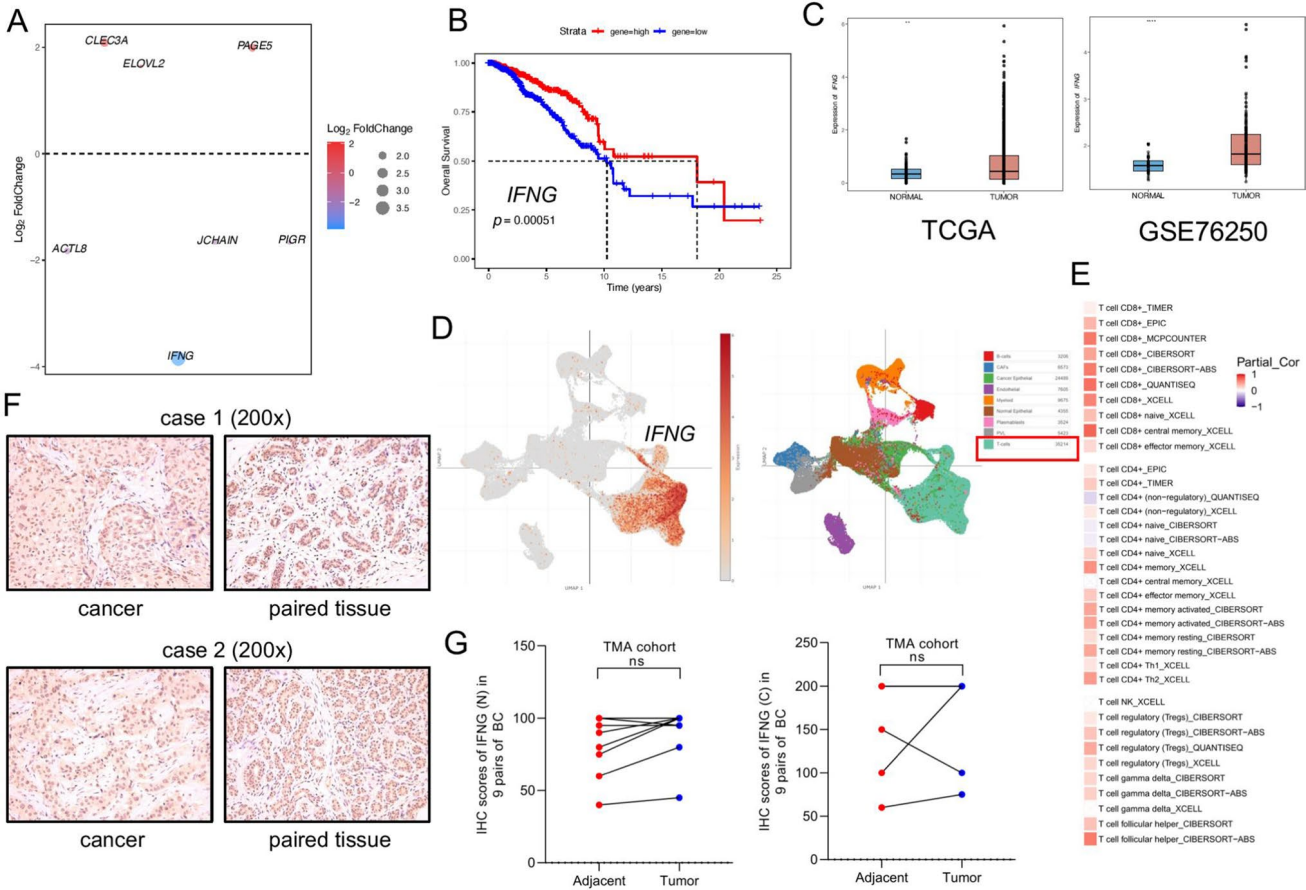
Analysis of *IFNG* in breast cancer. **A** The bubble diagram of gene expression changes; **B** Survival analysis: effect of *IFNG* expression on the survival of TCGA-BRCA; **C** The box plot: *IFNG* expression in different breast cancer datasets; **D** Single-cell sequencing of breast cancer: expression of *IFNG* among different cells in the tumor microenvironment (Single Cell Portal); **E** Relationship between *IFNG* and different T cell infiltration in the breast cancer tumor microenvironment (TIMER 2.0); **F** IFNG staining of paired samples in the tissue microarray (×200); **G** IFNG staining score of paired samples in TMA: the left side showing the nuclear staining score and the right side showing the cytoplasm staining score. ****P < 0.0001; **P < 0.01; *ns* no significance

Our study traced the differential expression changes of these seven crucial genes constructed in the signature between Cluster 1 and Cluster 3 (Fig. 8A). *IFNG* was most differentially changed in Cluster 1 and Cluster 3. Meanwhile, according to the above studies, *IFNG* was significantly positively correlated with the PD-1/PD-L1 pathway and immunotherapy response in breast cancer, so we focused on *IFNG*. In the TCGA dataset, the samples with high expression of *IFNG* had a better prognosis (*P* < 0.01) (Fig. 8B). The study further explored the distribution of *IFNG* expression in the samples. Expression of *IFNG* in breast tumor tissues was significantly increased in bulk samples of TCGA and GSE76250 (Fig. 8C). In the single-cell sequencing data (Fig. 8D), the distribution of expression of *IFNG* in the tumor was analyzed, and *IFNG* was mainly expressed by T cells in tumor tissues. Through TIMER2.0 (Fig. 8E), we analyzed the relationship between *IFNG* and the infiltration degree of various T cells (CD8+ T cell, CD4+ T cell, NK T cell, Tregs, γδ T cell, and T follicular helper cell) in the tumor microenvironment, indicating that *IFNG* was positively correlated with CD8+ T cell infiltration. At the same time, immunohistochemical staining was further performed on the breast cancer TMA (Fig. 8F). There were nine paired breast tissues to be evaluated, and there was no significant difference in the nuclear and cytoplasmic expression of IFNG between tumor cells and normal epithelial cells (Fig. 8G).

## Discussion

Breast cancer is a significant threat to women’s health, with substantial heterogeneity and few effective treatment drugs. In previous studies, the clinical subtype of breast cancer is the most important indicator to predict the effectiveness of immunotherapy, and triple-negative breast cancer, which has been reported as the most immunogenic subtype, only accounts for about 20% [39, 40]. However, immunotherapy has little effect on hormone receptor-positive patients, who account for 80% or more [12, 41], and the application of immunotherapy in breast cancer is minimal. Although some studies have predicted the immune efficacy of breast cancer patients through TMB, PD-L1 expression, tumor-infiltrating lymphocyte, or mismatch repair, there is still a big gap between them and their actual clinical application [9, 42].

PD-1 is mainly expressed in T cells, B cells, monocytes, or dendritic cells. As a transmembrane protein–ligand, PD-L1 on tumor cells is often up-regulated by 20% to 34% in breast cancer [43]. PD-L1 binds to PD-1 on the surface of immune cells to inhibit immune responses in the tumor microenvironment, leading to immune escape [7]. The specific mechanism of the PD-1/PD-L1 pathway in breast cancer has not been elucidated, and the characteristic genes have been studied relatively little. Therefore, it is urgent to evaluate and explore the PD-1/PD-L1 pathway and its characteristic immunological genes in breast cancer by multi-dimensional and multi-omics. In our study, the characteristic genes affecting the breast cancer PD-1/PD-L1 pathway were obtained by associating WGCNA with the PD-1/PD-L1 pathway. Based on this, molecular subtypes of breast cancer were explored. There are significant differences in each subtype's clinical and immunological characteristics, indicating that the PD-1/PD-L1 pathway can distinguish breast cancer subtypes, which has essential clinical guiding value.

Then, the prognostic signature was constructed according to genes related to PD-1/PD-L1 pathway molecular typing. The C-index of the TCGA training set was 0.747, and the C-index of the nomogram was 0.812. We first conducted the RiskScore assessment on different molecular subtypes of breast cancer, and the results also suggested that the signature constructed by us could better predict the prognosis of each subtype. Many scholars have proposed signatures of their related research from different perspectives. Wang et al. [44] constructed a prognostic signature consisting of nine ferroptosis-related genes (AUC = 0.618, 0.653, and 0.663 at 1-, 2- and 3-year, respectively). Li et al. [45] established a prognostic signature related to macrophage marker genes (AUC = 0.662 and 0.701 at 3- and 5-year, respectively). He et al. [46] created the glycolysis signature to predict the survival time of breast cancer patients, and the AUC of the training set was 0.719. Compared with previous related studies, our study improves reliability through multiple validations of external databases. The prediction ability of this signature is better than that of these published signatures. Our nomogram shows a higher C-index. The prognostic signature constructed by the PD-1/PD-L1 pathway molecular typing in this study may improve the predictive ability of breast cancer. On the other hand, our study still has some limitations, and the prognostic signature needs further clinical validation.

The biomarkers obtained by integrated multi-omics analysis of tumors often suggest clinical precision medicine [47]. We observed that breast cancer patients with different RiskScores had significantly different IC_50_ values for common clinical antitumor drugs, which provided the direction for subsequent precision drug use. By observing the mutation spectrum of breast cancer samples with different RiskScores, it was also found that there were significant differences in gene mutations among different RiskScore samples. We further analyzed the tumor microenvironment between different RiskScore groups, and it was also apparent that the expression of *PDCD1*, *CD274*, *CTLA-4*, *HAVCR2* and *LAG3* in the low-RiskScore group was relatively high. The RiskScore constructed based on the PD-1/PD-L1 pathway influenced the degree of infiltration of multiple cells in the breast cancer tumor microenvironment and further influenced the response of breast cancer patients to immunotherapy. The low-RiskScore group had a higher immunotherapy score and this suggested that patients with low RiskScores might be more likely to benefit from immunotherapy. In breast cancer, PD-1/PD-L1 may affect various signaling pathways, such as PTEN/PIK3CA, ERBB2 and STAT3, affecting various biological processes of tumor cells and the interaction between tumor cells and immune cells [48]. Similarly, this study also profoundly explored the possible mechanism of different RiskScore causing different biological behaviors. We hypothesize that in high-RiskScore breast cancer, the suppression of inflammation and apoptosis and the promotion of abnormal energy metabolism such as glycolysis may lead to the reduction of CD8+ T cells, M1 macropahge and activated NK cells in the tumor microenvironment of breast cancer.

Our study constructed the prognostic signature based on breast cancer PD-1/PD-L1 pathway molecular typing-related genes (*IFNG*, *JCHAIN*, *ELOVL2*, *PIGR*, *PAGE5*, *ACTL8*, and *CLEC3A*). These key genes were the highlight of the study. Interestingly, in previous experimental studies, some of these genes have been suggested to play an essential role in breast cancer. In the subgroup analysis of the NeoSphere trial, high *IFNG* expression was associated with a pathological complete response in the breast [49]. Knockdown of *ELOVL2* induced reprogramming of lipid metabolism in breast cancer and contributed to its malignant phenotype [50]. The increased expression of *PIGR* in breast cancer might reflect the polarization of tumor-associated immune cells [51]. *ACTL8* was up-regulated in triple-negative breast cancer and was associated with poor prognosis [52]. Patients with breast invasive ductal carcinoma with high *CLEC3A* expression were associated with higher lymph node metastasis and poor overall survival [53]. We analyzed the correlation between signature genes and the PD-1/PD-L1 pathway in breast cancer and the efficacy of immunotherapy in patients, as well as the differential expression of signature genes in samples. Our results suggested that *IFNG* was the most promising predictor we found and was most closely related to immunotherapy in breast cancer patients. It pointed out the direction for our follow-up research.

*IFNG*, a gene encoding a kind of cytokine in the type II interferon family, is mainly expressed by immune system cells and plays a vital role in immune monitoring in the tumor microenvironment [54]. Several studies have revealed a link between *IFNG* and breast cancer. In triple-negative breast cancer, tumors with high CD8 scores have abundant interferon-α and interferon-γ response and have more anti-cancer immune cell infiltration [55]. *IFNG* is a driver of the association between NK cells and clinical response to trastuzumab in patients with HER2-positive breast cancer [56]. The clinical surgical specimens of triple-negative breast cancer patients after neoadjuvant chemotherapy were detected. The expression of *IFNG* in the pathologic complete response (pCR) group was higher than that in the non-pCR group [57]. Tumor tissue contains not only tumor cells, but also a variety of infiltrating immune cells, and the bulk RNA-seq technique could not distinguish whether gene expression in a sample was due to a specific cell class. The expression of *IFNG* detected was the total number of cells in samples. Therefore, we further analyzed the expression of IFNG in tumor cells and normal mammary epithelial cells in paired breast cancer samples in the TMA, and the immunohistochemical results showed that there was no significant difference in the expression of IFNG between tumor cells and normal mammary epithelial cells. We speculated that the increased expression of *IFNG* might be caused by other components of non-tumor cells in the tumor microenvironment. Therefore, we further used the single-cell sequencing results to answer our questions. Single-cell sequencing indicated that *IFNG* was mainly expressed in T cells in the tumor microenvironment. Further analysis indicated that *IFNG* expression in tumor tissues was positively correlated with the degree of CD8+ T cell infiltration in breast cancer. Our results suggest that *IFNG* may be mainly expressed by CD8+ T cells in the breast cancer tumor microenvironment. Patients with high expression of *IFNG* have a better prognosis, which is consistent with *IFNG* as a protective factor in the risk signature.

In summary, our study conducted a multi-dimensional and multi-directional analysis of the characteristics of the breast cancer PD-1/PD-L1 pathway, and further constructed a prognostic signature. This study revealed the value of prognostic signature according to PD-1/PD-L1 pathway molecular typing in breast cancer clinical practice. The signature obtained in this study can evaluate the efficacy of clinical drug treatment and the prognosis of patients as a whole, which provides a new direction and reference for the development of subsequent clinical trials. In the subsequent clinical research or practice, researchers can construct a detection panel, detect the blood or tissue samples of patients, and screen the sensitive population for immunotherapy before treatment. Based on this, researchers can determine the survival risk of patients before treatment, screen out the people with poor prognosis in advance, and further switch treatment strategies. The relationship between the vital signature gene *IFNG* expression and CD8+ T cell infiltration in the breast cancer microenvironment was further analyzed. There are still some shortcomings in our study. Future research needs to explore the mechanism of *IFNG* in the breast cancer tumor microenvironment and actively promote the clinical application.

## Conclusion

The signature related to breast cancer PD-1/PD-L1 pathway molecular typing combined with clinical characteristics can more accurately predict patients’ 1-year, 3-year, and 5-year survival. *IFNG*, as a component gene in the prognostic signature, is closely related to CD8+ T cell infiltration in the breast cancer tumor microenvironment.

## Supplementary Information

Supplementary file 1

Supplementary file 2

## Data Availability

The public datasets used in this study can be queried and downloaded from public databases by corresponding numbers. If you have further questions, please contact the authors.
