# Supplementary material for: A novel PD-1/PD-L1 pathway molecular typing-related signature for predicting prognosis and the tumor microenvironment in breast cancer
Source: Discov Oncol. 2023 May 8;14:59. doi: 10.1007/s12672-023-00669-4 (PMC10167089; doi:10.1007/s12672-023-00669-4)
Supplement: Supplementary file 2 [file 12672_2023_669_MOESM2_ESM.docx]

**
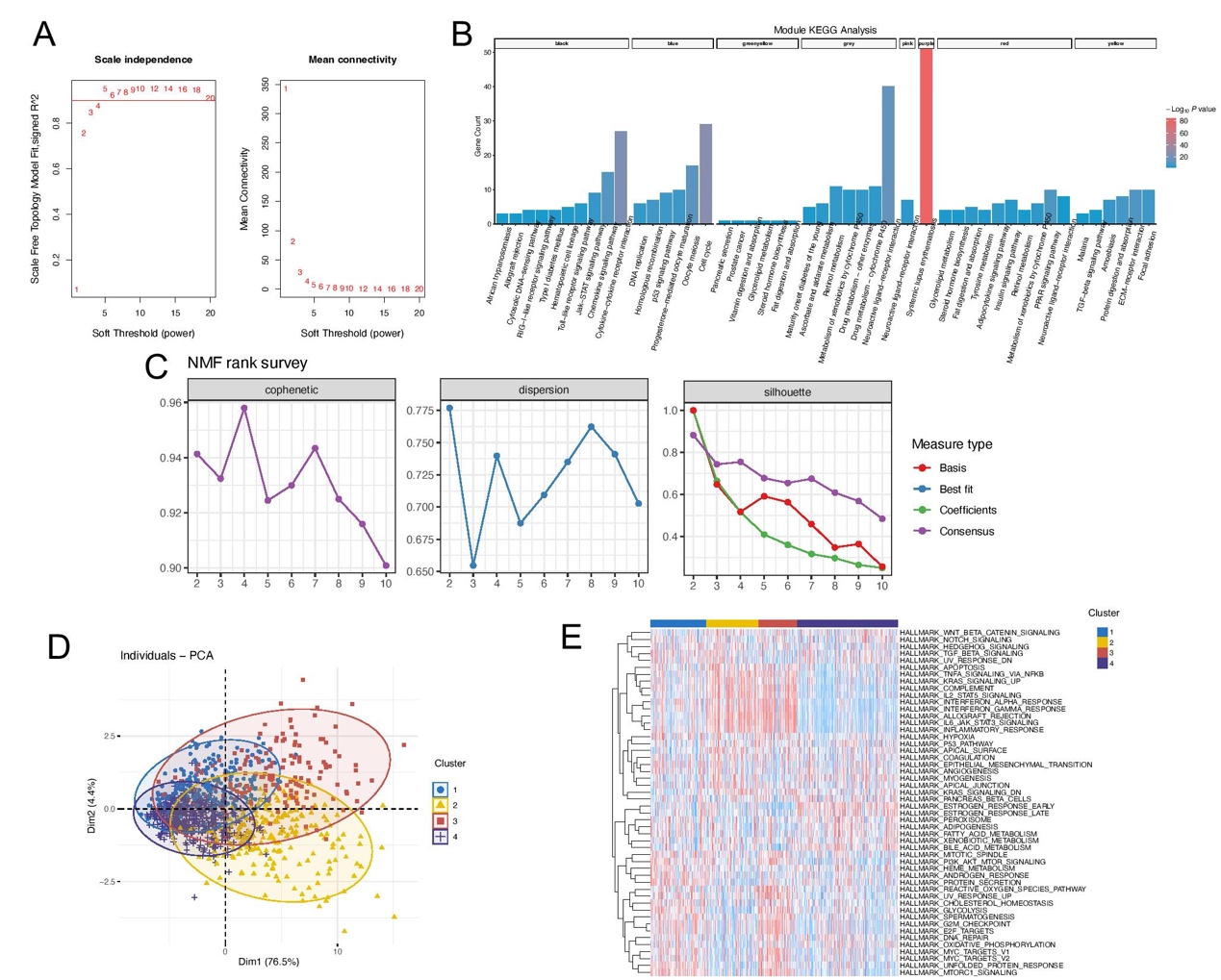
Fig. S1 (A)** Network topology analysis of soft threshold (1-20). The left panel showing that when the scale-free fitting index was 0.9, the minimum soft threshold was 4. The right panel showing the network connectivity under different soft thresholds; **(B)** KEGG analysis of modules; **(C)** The line chart of cophenetic correlation, dispersion and silhouette distribution with rank 2-10, At K = 4, there was a clear turning point; **(D)** The PCA plot of different subtypes; **(E)** Cancer hallmark pathway heatmap of different molecular subtypes of breast cancer in the TCGA database.

**
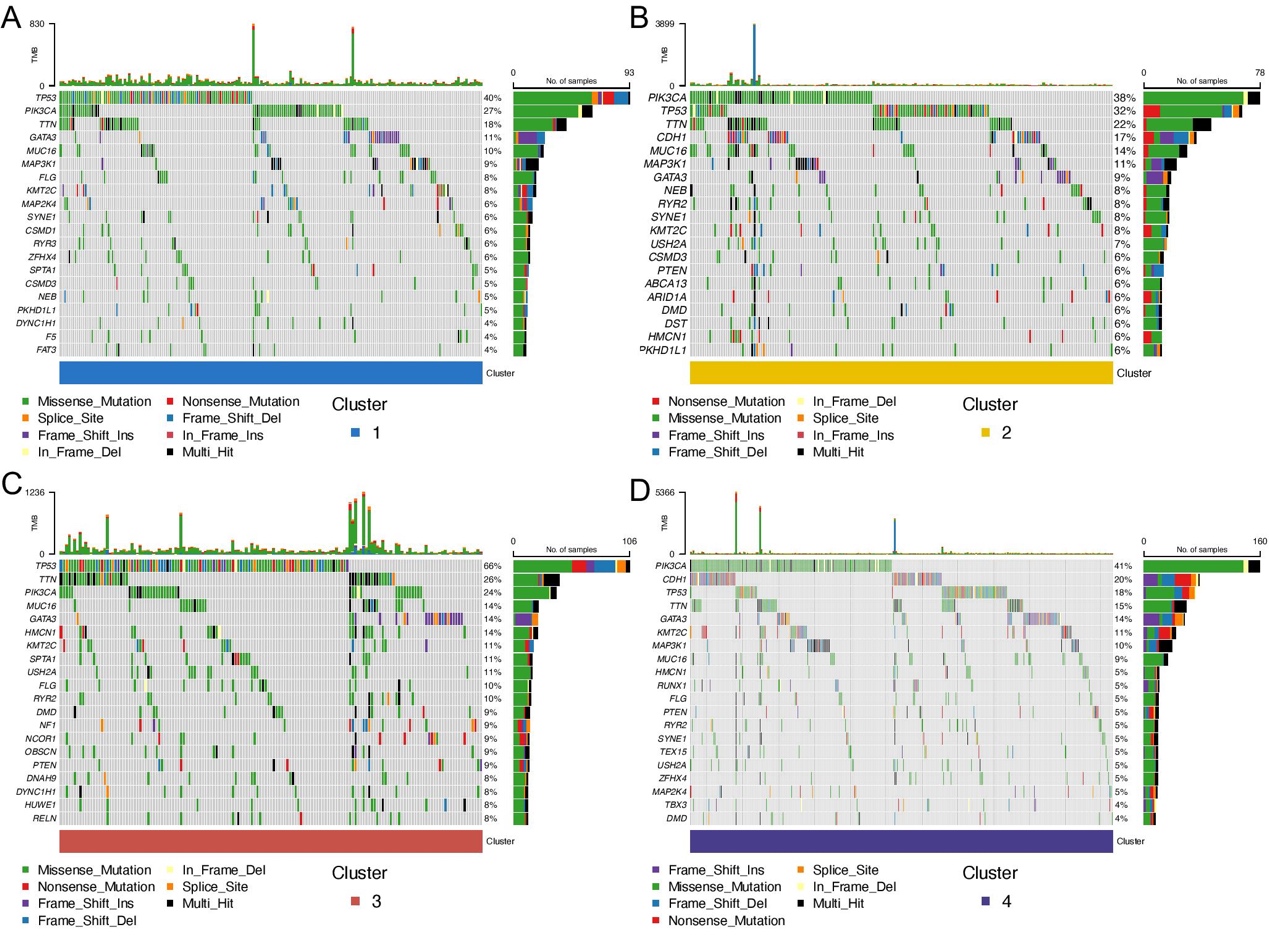
Fig. S2** The gene mutation waterfall diagram of different molecular subtypes of breast cancer in the TCGA database. **(A)** Cluster 1; **(B)** Cluster 2; **(C)** Cluster 3; **(D)** Cluster 4.

**
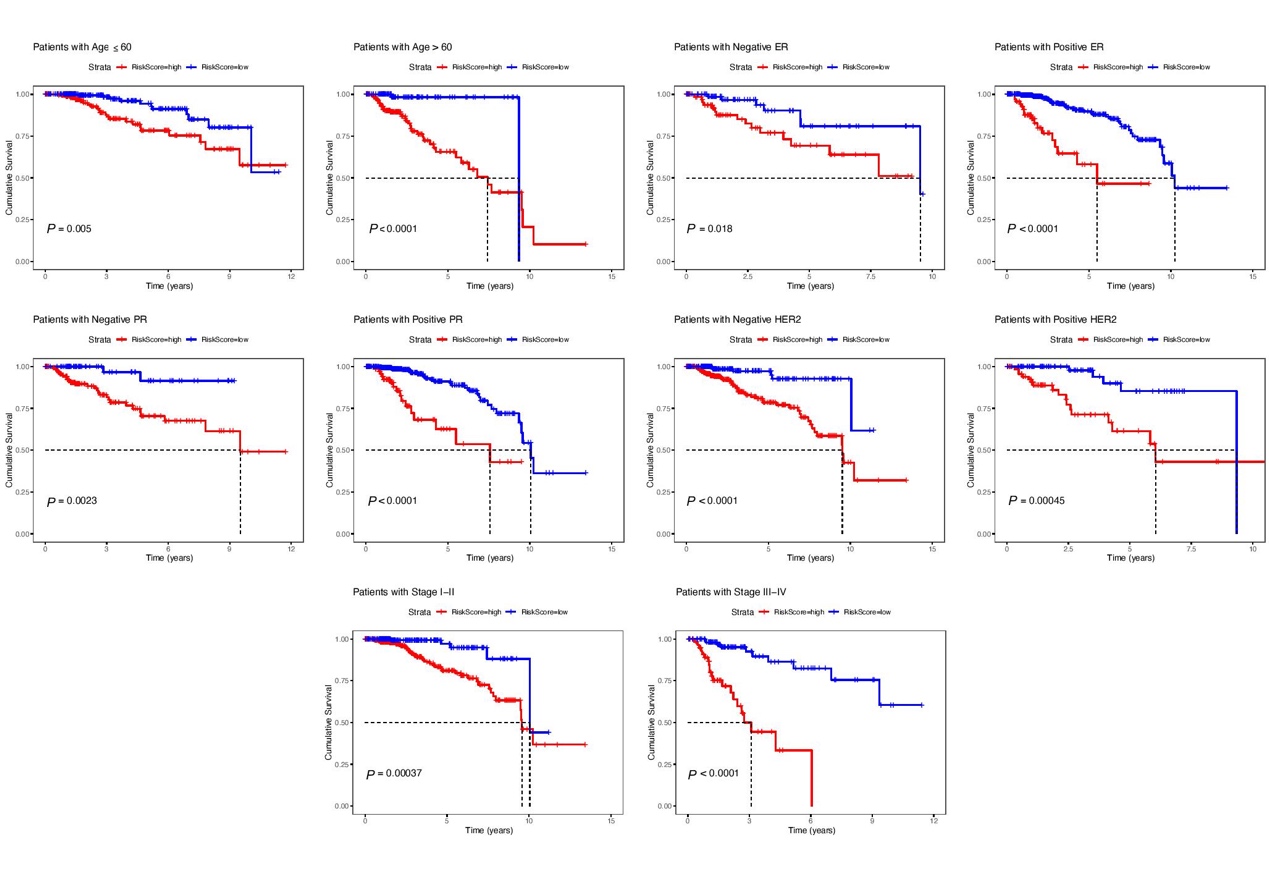
Fig. S3** The impact of the RiskScore on the survival of different age, ER/PR/HER2 status, and tumor stage in the TCGA-BRCA database.

**
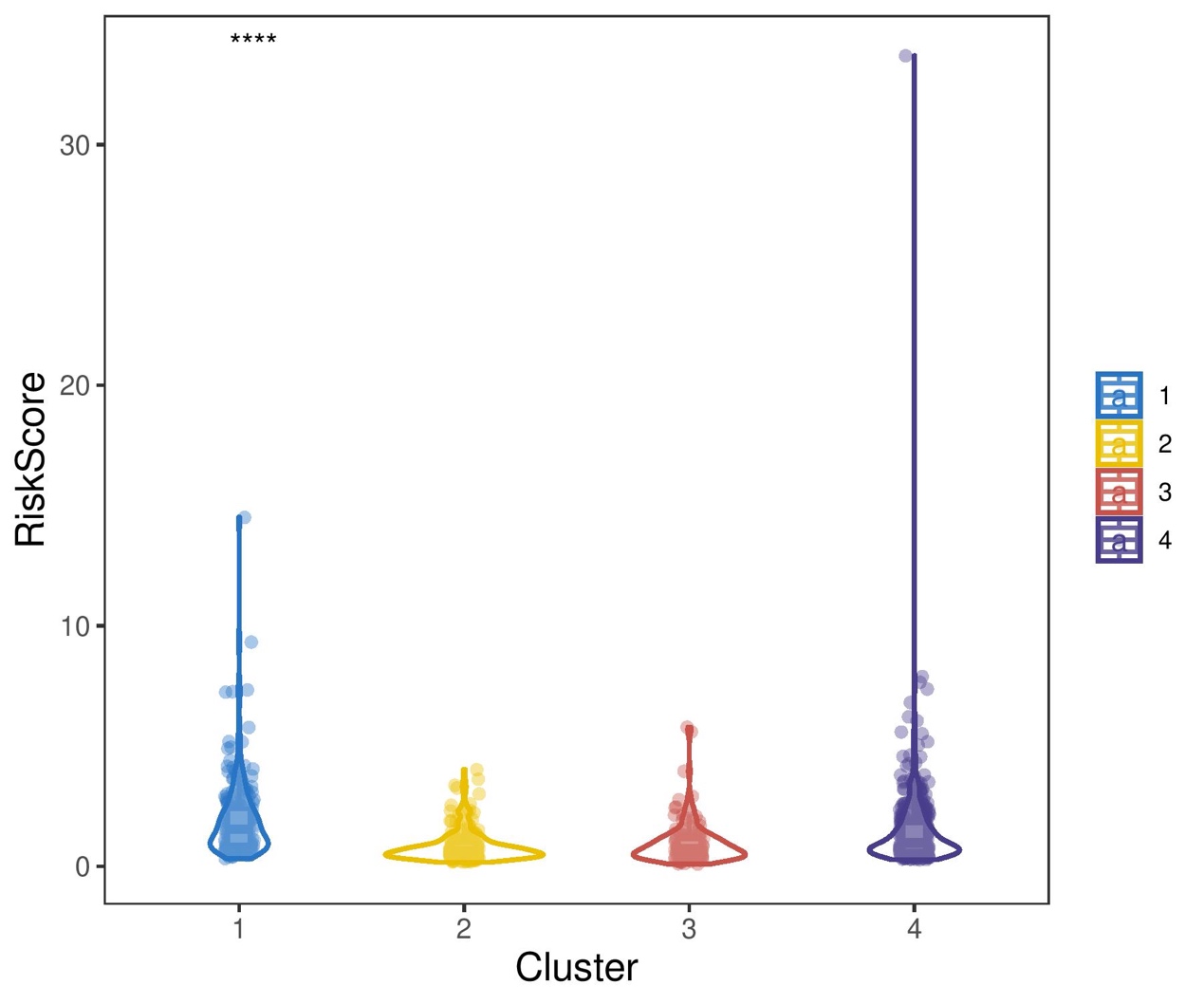
Fig. S4** Comparison of PD-1/PD-L1 pathway differences among different breast cancer molecular subtypes. *****P* < 0.0001.

**
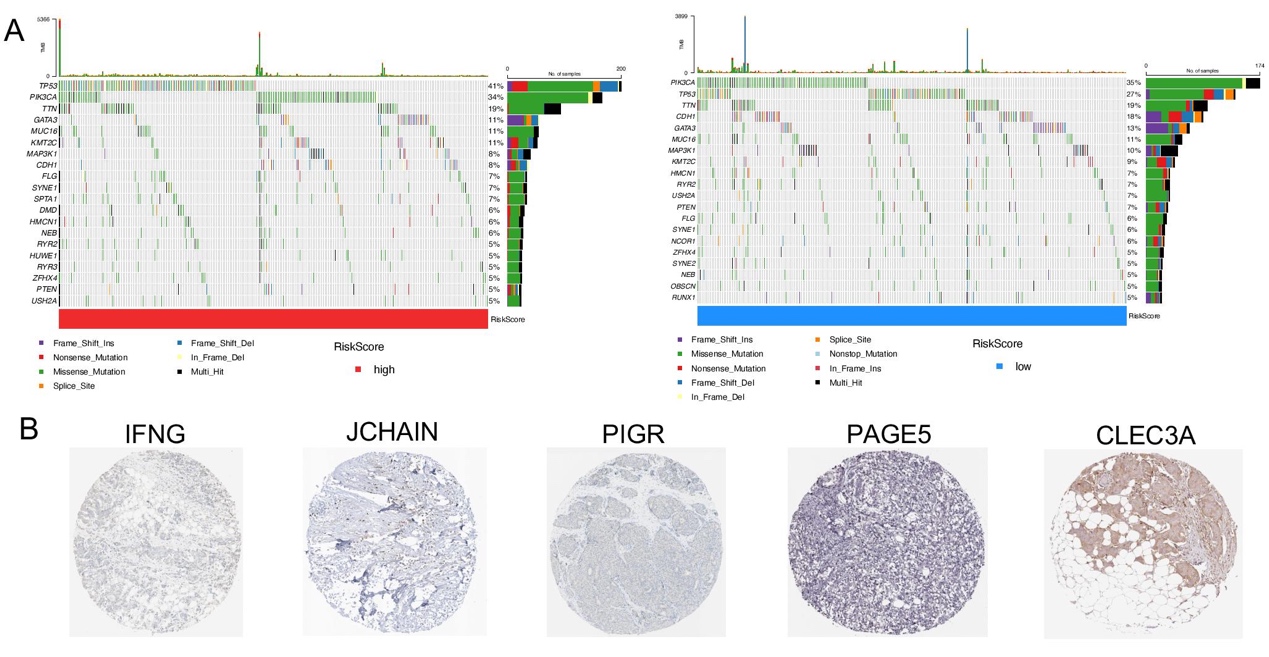
Fig. S5** Analysis of clinical characteristics of the prognostic signature. **(A)** Mutation waterfall heatmap of samples in patients with different RiskScore groups; **(B)** Immunohistochemical staining of IFNG, JCHAIN, PIGR, PAGE5, and CLEC3A in cancer tissues (HPA database).
